# Mating and Pathogenicity of the Dominant *Colletotrichum* Species Associated with Anthracnose Disease of Mango

**DOI:** 10.3390/jof11110762

**Published:** 2025-10-23

**Authors:** Rui Wang, Haoyue Cheng, Juan Shu, Suiping Huang, Lihua Tang, Tangxun Guo, Xiaolin Chen, Tom Hsiang, Qili Li

**Affiliations:** 1 Institute of Plant Protection, Guangxi Academy of Agricultural Sciences, Nanning 530007, China; wangrui20010504@163.com (R.W.); 18986335683@163.com (H.C.); suipinghuang@126.com (S.H.);; 2 Key Laboratory of Green Prevention and Control on Fruits and Vegetables in South China Ministry of Agriculture and Rural Affairs, Nanning 530007, China; 3 Guangxi Key Laboratory of Biology for Crop Diseases and Insect Pests, Nanning 530007, China; 4 School of Environmental Sciences, University of Guelph, Guelph, ON N1G 2W1, Canada; thsiang@uoguelph.ca

**Keywords:** *Mangifera indica*, *Colletotrichum*, sexual reproduction, pathogenicity

## Abstract

Anthracnose is one of the main diseases of mango, which seriously affects the yield and quality. Previous studies found that mango anthracnose in China involves at least 13 species of *Colletotrichum.* From mango with anthracnose symptoms samples, we previously obtained 134 strains from 13 species, and 26.0% were *C. fructicola*, while 27.6% were *C. siamense*, and 31.3% were *C. asianum*, with a few specimens for each of the remaining species. These three main species were used in intraspecific mating tests to assess sexual reproduction. The intraspecific mating tests revealed that *C. fructicola* and *C. siamense* readily produced ascospores, while *C. asianum* did not. From the 595 intraspecific crosses with 35 isolates of *C. fructicola*, 34 crosses were considered fertile. Among these, thirty single-ascospore isolates were chosen for pathogenicity testing and genetic variation analysis (ITS and ApMat loci). The results revealed that some progeny showed higher aggressiveness than their parents, while some progeny showed lower aggressiveness. Future tests are needed to assess the genetic basis of these aggressiveness differences. The results provide a scientific basis for further research on sexual reproduction and pathogenicity of *Colletotrichum*, which may allow for comprehensive disease prevention and control.

## 1. Introduction

Mango (*Mangifera indica* L.) is a nutritious tropical fruit of high economic value [1,2]. Mango is mainly cultivated in Hainan, Yunnan, Guangxi, Sichuan and other southern provinces in China, accounting for 11% of the world’s total output, where China is the second largest producer [3,4]. During mango cultivation, anthracnose is one of the most serious diseases, and epidemics will cause huge yield losses, even up to 100% [5,6]. At present, chemical control is the main method for mango anthracnose management during production and post-harvest [7]. However, the overuse of fungicides has led to environmental pollution and pesticide residues on foods [8].

The use of disease-resistant varieties is one of the economically feasible, effective and environmentally friendly measures to control anthracnose disease [9]. However, due to large number of mango varieties and wide range of susceptibility to anthracnose, plus the continuously evolving diversity of aggressiveness of *Colletotrichum* spp., originally highly resistant varieties may lose resistance, leading to widespread disease [10]. *Colletotrichum* is ranked eighth among the top ten economically important fungal pathogen groups, and has the diversity to adapt to various hosts [11]. Previous research in our lab has revealed that there are 13 species of *Colletotrichum* causing anthracnose on mango in China: *C. asianum*, *C. cliviae*, *C. cordylinicola*, *C. endophytica*, *C. fructicola*, *C. gigasporum*, *C. gloeosporioides*, *C. karstii*, *C. liaoningense*, *C. musae C. scovillei*, *C. siamense* and *C. tropicale* [12,13].

The genetic variation in the anthracnose pathogen can occur through different mechanisms, such as hyphal fusion, point mutations, and sexual reproduction [14,15,16,17,18]. In the definition of biological species, a group of individuals that can mate is considered to be of the same species [19]. The association between sexual and asexual forms may serve as a basis for identifying fungal species [20]. Sexual reproduction is presumably a critical element for generating genetic recombination and aiding in pathogen population evolution [21]. Mating type loci are the genetic determinants of sexual affinity, and have an important influence on the evolution of genetic variation and control of fungi [22]. Unlike many other genera, species of *Colletotrichum* can contain both homothallic and heterothallic individuals, and cross fertility among isolates within a species can range from absolutely sterility to high fertility [23]. Currently, species such *C. graminicola*, *C. fioriniae*, *C. gloeosporioides* and *C. fructicola* have been described as demonstrating both homothallism and heterothallism [18,24,25,26]. In these species, some isolates are capable of sexual reproduction when cultured alone, while other isolates require a mating partner in order to produce sexual offspring [18,24,25,26]. In *C. fructicola*, this behavior is thought to be facilitated by a positive/negative switch, with both isolates able to mate with each other, but with a preference for cross-mating during mycelial interactions [18]. However, this system is not well understood, and within a single species of homothallic and heterothallic isolates, it is difficult to accurately assess species mating type, gender identity, and mating compatibility. Therefore, investigation of the sexual reproduction processes and occurrence in *Colletotrichum* spp. is of great significance for a fuller understanding of the role of sexual reproduction in epidemics caused by *Colletotrichum* spp.

The purpose of this study was to evaluate ascospore production by examining intraspecific mating within three dominant species causing mango anthracnose, and furthermore, to select ascospore progeny for aggressiveness compared to their parents. Examination of the sexual reproduction of *Colletotrichum* not only helps to reveal their genetic diversity, evolutionary mechanisms, and patterns of disease epidemics, but also provides an important theoretical foundation and practical guidance for disease control, taxonomy, and ecological research. Such results may facilitate better understanding and management of diseases caused by species of *Colletotrichum*.

## 2. Materials and Methods

### 2.1. Fungal Materials and Culture Media

A total of 144 strains were isolated and stored in the laboratory [13] for subsequent testing, and detailed information is provided in Table 1 and Table S1. Potato dextrose agar (2%) was supplemented with salts (2 g/L NaNO_3_, 1 g/L KH_2_PO_3_, 0.5 g/L MgSO_4_, 0.01 g/L FeSO_4_, and 0.5 g/L KCl) for culturing these fungi.

### 2.2. Sexual Compatibility Testing

For intraspecific mating tests, multiple isolates of the three dominant species causing mango anthracnose were selected: *C. asianum* (42 isolates), *C. siamense* (37 isolates) and *C. fructicola* (35 isolates). Following the methods of Cai et al. [27], the autoclaved culture medium was poured into Petri dishes, and flat wooden toothpicks were added to serve as a substrate for ascomata formation. Three sterilized toothpicks were arranged in an “N” shape in each plate. Two 9-mm-diamter hyphal plugs were placed 1 cm to the left and the right sides of the “N”, and incubated at 25 °C under constant light. The plugs were taken from the growing edges of two parental isolates incubated on PDA at 25 °C for 7 days. After 30 days, the mating plates were examined under a microscope for the presence of ascomata. Successful crosses resulted in a ridge of mature ascomata formed at the line of contact. Ascomata were removed from the toothpicks and mounted in drops of sterilized water and crushed under cover slips. Each cross was replicated three times.

**Figure 1:**
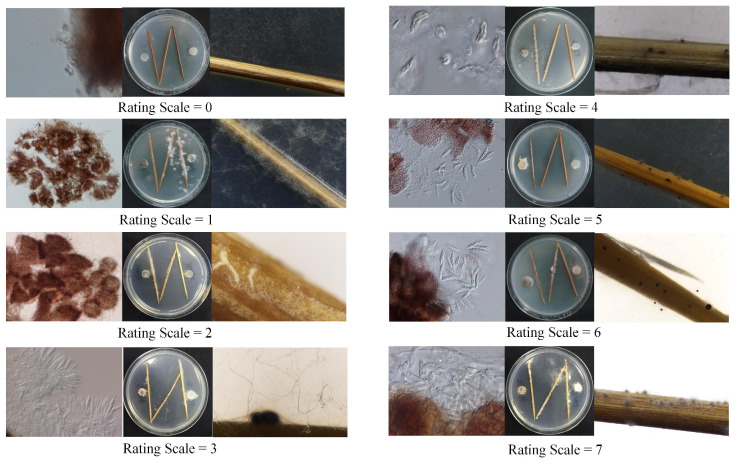
Rating system for intra-specific crosses, showing examples of each rating scale from 0–7.

For each cross, a rating system was used to score the sexual reproduction on a scale of 0–7 [25]: 0 = no structures observed at the zone of colony interaction on the center toothpick where the two colonies merged; 1 = small sterile structures (possibly protoperithecia); 2 = sterile perithecia with beaks, no asci; 3 = sterile perithecia with asci, no ascospores; 4 = asci with a few ascospores; 5 = fertile perithecia with many ascospores, few asci with eight spores; 6 = fertile perithecia with abundant ascospores, many asci with eight spores; 7 = perithecia exuding ascospores from ostiole. Crosses scoring 4 or higher were considered fertile (Figure 1).

### 2.3. Genetic Variation Among Progeny of C. fructicola Crosses

After the successful mating of parental isolates to produce ascospores, the asci were placed in water droplets, crushed with a cover glass, and the ascospores were dilution-plated on PDA plates for single-spore isolation. From 15 intraspecific crosses of *C. fructicola*, two progenies each (totaling 30) were selected for sequencing of the ITS and ApMat to verify that they matched *C. fructicola*, and to assess genetic diversity. Parental and progeny isolates are listed in Table 1. The genomic DNA of each isolate was extracted using DNAsecure Plant Kit (TIANGEN Biotech, Beijing, China). PCR with a total volume of 25 µL was composed of 11.5 µL of Premix Ex Taq (Takara Bio, Dalian, Liaoning, China), 10.5 µL of sterile distilled water, 1.0 µL of DNA template, and 1.0 μL each of the forward and reverse primers (10 mM). PCR targeting the ITS used the universal primers ITS1 (TCCGTAGGTGAACCTGCGG)/ITS4 (TCCTCCGCTTATTGATATGC) [28], and for the ApMat region, the primers were AM-F (TCATTCTACGTATGTGCCCG)/AM-R (CCAGAAATACACCGAACTTGC) [29]. PCR cycling conditions included 95 °C for 5 min, 35 cycles of 95 °C for 30 s, 55 °C (for ITS) or 62 °C (for ApMat) for 30 s, and 72 °C for 1 min; and a final extension at 72 °C for 10 min. The PCR products were visualized on 1% agarose gel electrophoresis, and the PCR products were sent to Sangon Biotech (Shanghai, China) for sequencing.

The consensus sequences of forward and reverse primers for each isolate were assembled and edited using DNAMAN v.5.2.2 (Lynnon Biosoft). The sequencing results were analyzed using BLAST against NCBI database (https://www.ncbi.nlm.nih.gov, accessed on 15 July 2025). Then, extype or ex-epitype isolates were selected from NCBI as references. MUSCLE [30] was used to generate sequence alignments for each marker. For species identification, concatenated sequences of two markers (ITS and ApMat) were used. The sequence differences between isolates were assessed in multiple sequence alignment using MUSCLE [30].

### 2.4. Assessment of Aggressiveness of Sexually Produced Progeny of C. fructicola on Mango Fruit

The monoascospore offspring of 30 different isolates of *C. fructicola* were used in pathogenicity tests with two isolates per cross. In addition, the 17 parental isolates were also used in pathogenicity tests. Healthy mango fruits were disinfected with 75% alcohol for 10 s and 2% sodium hypochlorite solution for 1 min, washed three times using sterile water, and dried on sterilized filter paper. Multiple light punctures in a 5-mm-diameter circle were made with a sterilized toothpick on detached healthy fruits, followed by inoculation with one 5-mm-diameter hyphal plug per wound site, while control fruits were each mock inoculated with a 5-mm-diameter PDA plug. Then the fruits were placed in a plastic box lined with wet filter paper and sealed and incubated at 28 °C. Symptoms were observed daily up to 7 days, and the lesion diameters were measured in two directions to evaluate aggressiveness.

### 2.5. Statistical Analyses

Data Processing System software v. 3.01 [31] was used for variance analysis of lesion diameters. If there were significant treatment effects (*p* ≤ 0.05), LSD (test of least significant difference) was used to separate the means at *p* = 0.05.

## 3. Results

### 3.1. Mango Anthracnose Intraspecific Isolate Mating

Forty-two isolates of *C. asianum* were used in intraspecific testing resulting in 861 crosses. None of the crosses produced ascospores, and only 28 crosses produced sterile structures (Table 2). Thirty-seven isolates of *C. siamense* were used in intraspecific testing resulting in 666 crosses (Table 2). Only 11 of these crosses were considered fertile. These 11 crosses mainly involved 16 isolates (Table S3). Thirty-five isolates of *C. fructicola* were used in intraspecific testing resulting in 595 crosses, and only 34 of these were considered fertile crosses, with rating class over 4 (Table 2). These 34 fertile crosses mainly involved 26 isolates (Table S4).

### 3.2. Genetic Variation Among Progeny of C. fructicola Crosses

Isolates were subjected to ITS and ApMat sequencing. Combining ITS and ApMat sequence data gave a total length of 1420 bp (containing gaps created during the alignment process, ITS: 0-536, ApMat: 537-1420). We used *C. fructicola* (ICMP18581, type species) as a reference isolate, and compared all sequences to it to assess loci changes. There were some single nucleotide polymorphisms: five isolates had one site difference; two isolates had two; eight isolates had three; 11 isolates had four; three isolates had five; and one isolate had seven. However, over 99.5% of the 1420 bp length was conserved among all isolates. In addition, during the comparison of the sequences with the parental isolates, a total of 15 offspring had site variations in the ApMat gene (the same site was different from both the parents), among which 7 isolates (MG3-1, MG6-1, MG7-1, MG7-2, MG10-1, MG11-2 and MG12-1) had 1 site variation, 7 (MG3-2, MG4-2, MG6-2, MG8-1, MG8-2, MG13-1 and MG13-2) had 2 site variations, and 1 (MG14-1) had 3 site variations (Table S5). There was no variation in the ITS sequence region.

### 3.3. Aggressiveness of Sexually Reproduced Offspring

Based on intraspecific mating tests that identified 34 fertile crosses (rating class > 4) out of 595 involving 35 *C. fructicola* isolates, 15 crosses were randomly chosen for further study. The parental isolates of these 15 combinations showed differences in aggressiveness. Two offspring were selected from each mating experiment and compared with their parental isolates to assess their aggressiveness. There were three isolates with significantly increased aggressiveness compared to their parents. Five of them showed significant decreased aggressiveness compared to their parents (Table 3).

## 4. Discussion

We conducted intraspecific mating of the three dominant *Colletotrichum* species (*C. fructicola*, *C. siamense* and *C. asianum*) found on mango to assess ascospore production. In the rating system, crosses scoring 4 or higher were considered fertile. Under laboratory conditions, 43.2% of *C. siamense* isolates and 76.5% of *C. fructicola* isolates were observed to be interfertile, which is consistent with previous research [18,32], while not for *C. asianum*. *C. asianum* produces an ɑ-factor protein harboring six mature repeats, made up of four different amino acid sequences. This suggests that this region is rapidly evolving and may contribute to the unpredictable nature of mating in this genus [33]. Sexual reproduction in fungi is a very complex cellular process that takes place under specific environmental and genetic conditions necessary for the expression of the genes regulating the process [34]. There may be unseen sexual stages or incompatible mating types between two isolates [35], or random events of heterokaryon formation and nuclear coordination [36], or genetic recombination and adaptive changes [37], and many other possibilities that prevent successful mating. Future studies should combine molecular markers, gene knockout and microscopic observation to clarify specific mechanisms. Whether specific pairings produce viable offspring is also dependent on the environment. Some hybrids may be fully viable and fertile in a benign environment, such as under lab conditions, but unfit in a natural environment. This can be the case if hybrids display intermediate traits between parental phenotypes, and are poor competitors in either parental environment [38]. We did not find fertile crosses among 42 isolates of *C. asianum*, which implies that the isolates we used were not homothallic, but the conditions may not have been appropriate for homothallic or heterothallic reproduction. For example, some specific nutrient or substance might be needed for homothallic reproduction, and we do not know if two different mating types were present among these isolates needed for heterothallic reproduction.

Because of the differential aggressiveness of the parental isolates, the 30 progeny produced by several intraspecific matings of *C. fructicola* isolates had varied aggressiveness to mango. However, eight isolates were significantly different from their parents (two progeny showed higher aggressiveness than their parents, while six progeny showed lower aggressiveness). In some fungal species, pathogenicity and mating are linked and mating is considered an important step in the lifecycle. For instance, in the smut fungus *Ustilago maydis*, the dikaryotic structures required for the growth of the fungus are the result of mating, and if mating does not take place, the organism remains avirulent [39]. At the genetic level, there are various genes, proteins and metabolic pathways that regulate the sexual process in fungal species. The genes harbored at the mating type (MAT) locus are the major regulators of sex in ascomycete fungi, and determinants of the mating strategies, and are thus part of the mating or breeding systems [34]. Depending on the fungal species, mating compatibility and the occurrence of sexual reproduction can be determined by a single site of one of the two mating types, or by a double site with more than two specific mating types [40]. The proteins encoded by mating type genes are mostly regulatory factors located in the upstream of the regulatory pathway of sexual reproduction, and are involved in the regulation of the recognition and fusion between cells of different mating type isolates, meiosis and the production of sexual spores, as well as the regulation of pheromones and their receptors [41,42,43]. It has for instance been shown that the pheromones and pheromone receptor of *Ustilago hordei* MAT-1 were necessary and sufficient to make *U. maydis* compatible with *U. hordei* MAT-2 [44]. In addition, the process of sexual reproduction is also affected by a variety of environmental factors, primary and secondary metabolism levels of fungal cells, and intracellular environmental balance conditions [45]. Therefore, in-depth research on the regulatory mechanism of fungal sexual reproduction is of great significance to reveal fully the variation and population diversity of *Colletotrichum*, as well as to develop targeted disease prevention and control strategies, and to provide key reference information for improving the effectiveness and prospect of disease resistance breeding.

In conclusion, intraspecific mating experiments demonstrated that both *C. fructicola* and *C. siamense* are capable of sexual reproduction, whereas *C. asianum* did not produce ascospores under the same conditions. From fertile crosses of *C. fructicola*, a subset of single-ascospore progeny exhibited significant variation in pathogenicity, with some isolates showing increased aggressiveness compared to their parents, while others were less aggressive. These findings not only confirm the occurrence of sexual reproduction in key *Colletotrichum* species but also highlight its potential role in generating pathogenic variability. Additionally, the genetic basis of aggressiveness differences among the sexual progeny of *C. fructicola* needs further investigation. Comparisons on completely sequenced genomes may provide insights into differences between sibling sexual progeny. These results provide a scientific basis for further research on sexual reproduction and pathogenicity of *Colletotrichum,* which may allow for comprehensive disease prevention and control.

## Figures and Tables

**Table 1:** Parental isolates of *C. fructicola* and two randomly selected ascospore offspring per cross used in pathogenicity tests.

Parental Isolates		Offspring 1	Offspring 2
GZ2-1	FJ29-1	MG1-1	MG1-2
GZ2-1	GZ19-1	MG2-1	MG2-2
GZ16	YN21-1-3	MG3-1	MG3-2
GZ21-2	YN21-1-3	MG4-1	MG4-2
GZ21-2	FJ25-1	MG5-1	MG5-2
HN19-1	FJ34-5	MG6-1	MG6-2
HN19-1	FJ25-1	MG7-1	MG7-2
HN19-1	FJ27-2	MG8-1	MG8-2
YN21-1-3	YN30-4	MG9-1	MG9-2
YN21-1-3	FJ27-2	MG10-1	MG10-2
YN43-1	FJ25-1	MG11-1	MG11-2
GZ2-1	FJ32-6	MG12-1	MG12-2
FJ13-3	FJ26-1	MG13-1	MG13-2
GZ14-1	HN7	MG14-1	MG14-2
GZ14-1	HN19-1	MG15-1	MG15-2

**Table 2:** Fertility rating of intraspecific mating tests involving many isolates for each of three species.

Species	Isolates	Number of Crosses in Each Fertility Rating Class (0 = Low, 7 = High)								Total
		0	1	2	3	4	5	6	7	
*C. asianum*	42	833	28	0	0	0	0	0	0	861
*C. siamense*	37	485	162	7	1	6	4	0	1	666
*C. fructicola*	35	476	71	12	2	5	20	7	2	595

**Table 3:** Differences in virulence on mango of parental isolates and two of their progeny from each cross in intraspecific mating of *C. fructicola*.

Group	Parent	Lesion Diameter (mm) *	Parent	Lesion Diameter (mm)	Offspring	Lesion Diameter (mm)	Offspring	Lesion Diameter (mm)
1	GZ2-1	6.1 ± 0.15 b **	FJ29-1	18.8 ± 1.63 a	MG1-1	16.5 ± 1.72 a	MG1-2	19.4 ± 2.27 a
2	GZ2-1	6.1 ± 0.15 c	GZ19-1	16.6 ± 0.94 ab	MG2-1	17.4 ± 1.73 a	MG2-2	12.2 ± 1.49 b
3	GZ16	15.4 ± 1.32 b	YN21-1-3	16.0 ± 1.04 b	MG3-1	32.2 ± 2.60 a	MG3-2	12.1 ± 0.94 b
4	GZ21-2	15.3 ± 0.82 ab	YN21-1-3	16.0 ± 1.04 a	MG4-1	9.9 ± 0.36 c	MG4-2	12.9 ± 0.61 b
5	GZ21-2	15.3 ± 0.82 bc	FJ25-1	19.8 ± 1.81 b	MG5-1	33.9 ± 3.96 a	MG5-2	8.4 ± 0.47 c
6	HN19-1	15.3 ± 0.60 a	FJ34-5	11.4 ± 0.58 b	MG6-1	6.5 ± 0.22 c	MG6-2	6.1 ± 0.22 c
7	HN19-1	15.3 ± 0.60 a	FJ25-1	19.8 ± 1.81 a	MG7-1	7.5 ± 0.62 b	MG7-2	8.3 ± 0.89 b
8	HN19-1	15.3 ± 0.60 b	FJ27-2	10.5 ± 0.56 c	MG8-1	7.8 ± 0.34	MG8-2	27.2 ± 2.38 a
9	YN21-1-3	16.0 ± 0.92 ab	YN30-4	13.2 ± 1.04 a	MG9-1	14.0 ± 0.70 ab	MG9-2	17.8 ± 1.66 b
10	YN21-1-3	16.0 ± 1.04 a	FJ27-2	10.5 ± 0.56 b	MG10-1	15.4 ± 1.36 a	MG10-2	14.4 ± 1.85 ab
11	YN43-1	9.4 ± 0.60 b	FJ25-1	19.8 ± 1.81 a	MG11-1	18.8 ± 1.04 a	MG11-2	19.4 ± 1.61 a
12	GZ2-1	6.1 ± 0.15 b	FJ32-6	18.5 ± 0.22 a	MG12-1	14.8 ± 0.97 a	MG12-2	13.5 ± 0.87 a
13	FJ13-3	8.3 ± 0.26 b	FJ26-1	17.6 ± 0.96 a	MG13-1	16.6 ± 1.21 a	MG13-2	18.1 ± 0.79 a
14	GZ14-1	37.6 ± 3.41 a	HN7	18.0 ± 0.97 b	MG14-1	12.4 ± 0.81 b	MG14-2	17.5 ± 1.18 b
15	GZ14-1	37.6 ± 3.41 b	HN19-1	15.3 ± 0.60 a	MG15-1	17.4 ± 0.70 a	MG15-2	17.8 ± 1.10 a

* Lesion diameter size of parental isolates and two of their progeny from each cross in intraspecific mating of *C. fructicola* were analyzed using LSD test with a one-way ANOVA by DPS v.7.05. ** Means followed by a letter in common were not significantly different at *p* ≤ 0.05.

## Data Availability

The original contributions presented in this study are included in the article/Supplementary Material. Further inquiries can be directed to the corresponding author.

## Supplementary Materials

The following supporting information can be downloaded at: https://www.mdpi.com/article/10.3390/jof11110762/s1, Table S1: Background information on the 134 tested *Colletotrichum* isolates from Mango. Table S2: Fertility rating of intraspecific mating tests involving many isolates for *C. asianum*. Table S3: Fertility rating of intraspecific mating tests involving many isolates for *C. siamense*. Table S4: Fertility rating of intraspecific mating tests involving many isolates for *C. fruticola*. Table S5: Sequence variation in the ITS and ApMat loci among *C. fructicola* parental isolates and their offspring.
