# Supplementary material for: Mating and Pathogenicity of the Dominant Colletotrichum Species Associated with Anthracnose Disease of Mango
Source: J Fungi (Basel). 2025 Oct 23;11(11):762. doi: 10.3390/jof11110762 (PMC12653461; doi:10.3390/jof11110762)
Supplement: Supplementary file 1 [file jof-11-00762-s001.zip › Table S1.pdf]

Table S1 Background information on the 134 tested *Colletotrichum* isolates from Mango

Isolates	Species	Geographic source	Variety	Isolation source	Date
FJ1-3	C. asianum	Senlingongyuan, Tianzhushan, Xiamen, Fujian	unknown	Leaf	2017/6/21
FJ4-2	C. asianum	Hongjiangzhongxue, Zhangpu, Fujian	unknown	Leaf	2017/6/21
FJ5-1	C. asianum	Rongjiang, Zhangpu, Fujian	Tainong	Leaf	2017/6/21
FJ6-4	C. asianum	Daling, Yunling, Yunxiao, Fujian	Tainong	Leaf	2017/6/21
FJ7-3	C. asianum	Nanshan, Puomei, Yunxiao, Fujian	unknown	Leaf	2017/6/21
FJ9-1	C. asianum	Daling, Yunling, Yunxiao, Fujian	unknown	Leaf	2017/6/21
FJ10-4	C. asianum	Nanshan, Puomei, Yunxiao, Fujian	wild-unknown	Leaf	2017/6/21
FJ11-1	C. asianum	Miyuanshengtainongchang, Qianting, Zhangpu, Fujian	Yuwen	Leaf	2017/6/21
FJ12-4	C. asianum	Miyuanshengtainongchang, Qiantign, Zhangpu, Fujian	Yuwen	Leaf	2017/6/21
FJ15-1	C. asianum	Miyuanshengtainongchang, Qiantign, Zhangpu, Fujian	Jinhuang	Leaf	2017/6/21
FJ18-3	C. asianum	Miyuanshengtainongchang, Qiantign, Zhangpu, Fujian	Jinhuang	Leaf	2017/6/21
FJ31-1	C. asianum	Gulou, Xiaoxi, Pinghe, Fujian	unknown	Leaf	2017/6/21
FJ31-6	C. asianum	Gulou, Xiaoxi, Pinghe, Fujian	unknown	Leaf	2017/6/21
FJ32-1	C. asianum	Guanshan, Changtai, Zhangzhou, Fujian	unknown	Leaf	2017/6/21
FJ33-2	C. asianum	Huangqin, Guoken, Zhangzhou, Fujian,	unknown	Leaf	2017/6/21
FJ36-6	C. asianum	Xintian, Banzhai, Pinghe, Fujian	unknown	Leaf	2017/6/21
FJ35-1	C. asianum	Wuzhai, Pinghe, Fujian	unknown	Leaf	2017/6/21
GD4-1	C. asianum	Dongan, Changqi, Huazhou, Guangdong	unknown	Leaf	2016/7/8
GD13-1	C. asianum	Shuanggang, Chikan, Zhanjiang, Guangdong	unknown	Leaf	2016/7/8
GD16	C. asianum	Xuwu, Nanting, Suixi, Zhanjiang, Guangdong	Tainong	Leaf	2016/7/8
GD27	C. asianum	Hongmaofeng,Wushi, Leizhou, Zhanjiang, Guangdong	Tainong	Leaf	2016/7/8
HN29-4	C. asianum	Haipo, Jiusuo, Ledong, Hainan	Tainong	Leaf	2017/4/17
HN34-1	C. asianum	Shilu, Changjiang, Hainan	wild-unkown	Leaf	2017/4/17
SC4-1	C. asianum	Shitou,Yakou, Miyi, Sichuan	unknown	Leaf	2016/9/8

SC19	C. asianum	Ashuda, Panzhihua, Sichuan	Kate	Leaf	2016/9/8
SC39	C. asianum	Zongfa, Renhequ, Panzhihua, Sichuan	Hongxiangya	Leaf	2016/9/8
YN2-1	C. asianum	Wuxin, Xinzhuang, Rongjiang, Huaping, Yunnan	Kate	Leaf	2016/9/6
YN3-1	C. asianum	Yeshishanzhuang, Xinzhuang, Rongjiang, Huaping, Yunnan	Kate	Leaf	2016/9/6
YN4-1	C. asianum	Wuxin, Xinzhuamg, Rongjiang, Huaping, Yunnan	Kate	Leaf	2016/9/6
YN6-1-1	C. asianum	Hejiawan, Xinzhuang, Rrongjiang, Huaping, Yunnan	Kate	Leaf	2016/9/6
YN7-1	C. asianum	Hejiawan, Xinzhuang, Rrongjiang, Huaping, Yunnan	Kate	Leaf	2016/9/6
YN11-2-1	C. asianum	Heai, Rongjiang, Huaping, Yunnan	Kate	Leaf	2016/9/6
YN12-2-1	C. asianum	Heai, Rongjiang, huaping, Yunnan	Kate	Leaf	2016/9/6
YN4-2-1	C. asianum	Wuxin, Xinzhuamg, Rongjiang, Huaping, Yunnan	Kate	Leaf	2016/9/6
YN18	C. asianum	Pingzi, Rongjiang, Huaping, Yunnan	Yingzui	Leaf	2016/9/6
YN19-1-1	C. asianum	Zheli, Rongjiang, Huaping, Yunnan	Yingzui	Leaf	2016/9/6
YN25-1-2	C. asianum	Hongxinfenchang, Namaluosizhai, Ganzhuang, Yuanjiang, Yunnan	Tainong	Leaf	2016/9/6
YN27-1-1	C. asianum	Hongxinfenchang, Namaluosizhai, Ganzhuang, Yuanjiang, Yunnan	Tainong	Leaf	2016/9/6
YN27-2-3	C. asianum	Hongxinfenchang, Namaluosizhai, Ganzhuang, Yuanjiang, Yunnan	Tainong	Leaf	2016/9/6
YN29-1	C. asianum	Hongxinfenchang, Namaluosizhai, Ganzhuang, Yuanjiang, Yunnan	Tainong	Leaf	2016/9/6
YN55-1	C. asianum	Yuanshisenlingongyuan, Xishuangbanna, Yunnan	unknown	Leaf	2016/9/6
YN56-2-1	C. asianum	Minzufengqingyuan, Xishuangbanna, Yunnan	Hongxiangya	Leaf	2016/9/6
FJ13-3	C. fructicola	Miyuanshengtainongchang, Qiantign, Zhangpu, Fujian	Yuwen	Leaf	2017/6/21
FJ17-5	C. fructicola	Miyuanshengtainongchang, Qiantign, Zhangpu, Fujian	Yuwen	Leaf	2017/6/21
FJ25-1	C. fructicola	Jinling, Yanxi, Changtai, Fujian	Jinhuang	Leaf	2017/6/21
FJ26-1	C. fructicola	Jinling, Yanxi, Changtai, Fujian	Jinhuang	Leaf	2017/6/21
FJ27-2	C. fructicola	Jinling, Yanxi, Changtai, Fujian	Jinhuang	Leaf	2017/6/21
FJ28-1	C. fructicola	Jinling, Yanxi, Changtai, Fujian	Guifei	Leaf	2017/6/21
FJ29-1	C. fructicola	Jinling, Yanxi, Changtai, Fujian	Guifei	Leaf	2017/6/21
FJ32-6	C. fructicola	Guanshan, Changtai, Zhangzhou, Fujian	unknown	Leaf	2017/6/21

FJ34-5	C. fructicola	Zhelin, Longxin, Nanzhao, Pinghe, Fujian	unknown	Leaf	2017/6/21
FJ35-5	C. fructicola	Xintian, Banzhai, Pinghe, Fujian	unknown	Leaf	2017/6/21
GZ19-1	C. fructicola	Tianfang, Bajie, Xingyi, Guizhou	Tainong	Leaf	2016/6/28
GZ1-2	C. fructicola	Bawei, Zhexiang, Wangmo, Guizhou	Tainong	Leaf	2016/6/28
GZ2-1	C. fructicola	Zhexiang, Wangmo, Guizhou	Tainong	Leaf	2016/6/28
GZ4	C. fructicola	Zhexiang, Wangmo, Guizhou	Tainong	Leaf	2016/6/28
GZ6-3	C. fructicola	Zhexiang, Wangmo, Guizhou	Tainong	Leaf	2016/6/28
GZ8-3	C. fructicola	Zhexiang, Wangmo, Guizhou	Tainong	Leaf	2016/6/28
GZ9	C. fructicola	Zhexiang, Wangmo, Guizhou	Tainong	Leaf	2016/6/28
GZ10	C. fructicola	Bayan, Suxiang, Wangmo, Guizhou	unknown	Leaf	2016/6/28
GZ12-1	C. fructicola	Wangmo city, Guizhou	Tainong	Leaf	2016/6/28
GZ13-1	C. fructicola	Qiaojiang, Youmai, Wangmo, Guizhou	Tainong	Leaf	2016/6/28
GZ14-1	C. fructicola	Qiaojiang, Youmai, Wangmo, Guizhou	unknown	Leaf	2016/6/28
GZ15-1	C. fructicola	Qiaojiang, Youmai, Wangmo, Guizhou	Tainong	Leaf	2016/6/28
GZ16	C. fructicola	Qiaojiang,Youmai, Wangmo, Guizhou	Tainong	Leaf	2016/6/28
GZ19-G-2	C. fructicola	Tianfang, Bajie, Xingyi, Guizhou	Tainong	Leaf	2016/6/28
GZ21-2	C. fructicola	TianZhai, Bajie, Xingyi, Guizhou	Tainong	Leaf	2016/6/28
GZ23-2	C. fructicola	Bannong,Yanjia, Ceheng, Guizhou	Hongxiangya	Leaf	2016/6/28
GZ25-1	C. fructicola	Bannong, Yanjia, Ceheng, Guizhou	unknown	Leaf	2016/6/28
HN7	C. fructicola	Nanxingnongchang, Yalongwan, Sanya, Hainan	Tainong	Leaf	2017/4/17
HN19-1	C. fructicola	Nanbinnongchang, Yacheng, Sanya, Hainan	Tainong	Leaf	2017/4/17
HN47-2	C. fructicola	Malong, Datian, Dongfang, Hainan	Tainong	Leaf	2017/4/17
HN54-1	C. fructicola	Baoying, Tianya, Sanya, Hainan	Tainong	Leaf	2017/4/17
YN13-1	C. fructicola	Longtou, Rongjiang, Huaping	Tainong	Leaf	2016/9/6
YN21-1-3	C. fructicola	hongxinfenchang, Namaluosizhai, Ganzhuang, Yuanjiang, Yunnan	unknown	Leaf	2016/9/6
YN30-4	C. fructicola	Ganba, Ganzhuang, Yuanjiang, Yunnan	unknown	Leaf	2016/9/6

YN43-1	C. fructicola	Minzufengqingyuan, Chengdong, Xishuangbanna, Yunnan	Yaomang	Leaf	2016/9/6
GD6-2	C. siamense	Ganbiao, Xinan, Huazhou, Guangdong	unknown	Leaf	2016/7/8
GD7-1	C. siamense	South Subtropical Crop Research Institute, China Academy of Tropical Agricultural, Guangdong	Tainong	Leaf	2016/7/8
GD8-1	C. siamense	South Subtropical Crop Research Institute, China Academy of Tropical Agricultural, Guangdong	Tainong	Leaf	2016/7/8
GD10-1	C. siamense	South Subtropical Crop Research Institute, China Academy of Tropical Agricultural, Guangdong	Tainong	Leaf	2016/7/8
GD11-1	C. siamense	South Subtropical Crop Research Institute, China Academy of Tropical Agricultural, Guangdong	Tainong	Leaf	2016/7/8
GD12-1	C. siamense	South Subtropical Crop Research Institute, China Academy of Tropical Agricultural, Guangdong	Tainong	Leaf	2016/7/8
GD14-2	C. siamense	Chikan, Zhanjiang, Guangdong	unknown	Leaf	2016/7/8
GD17	C. siamense	Nanting, Suixi, Zhanjiang, Guangdong	unknown	Leaf	2016/7/8
GD29-1	C. siamense	Hongmaofeng, Wushi, Leizhou, Zhanjiang, Guangdong	Danmang	Leaf	2016/7/8
GD29-2	C. siamense	Hongmaofeng, Wushi, Leizhou, Zhanjiang, Guangdong	Danmang	Leaf	2016/7/8
GD30-1	C. siamense	Hongmaofeng, Wushi, Leizhou, Zhanjiang, Guangdong	Tainong	Leaf	2016/7/8
GD30-2	C. siamense	Hongmaofeng, Wushi, Leizhou, Zhanjiang, Guangdong	Tainong	Leaf	2016/7/8
GZ11-1	C. siamense	Yangganshi, Sujiangxiang, Wangmo, Guizhou	unkown	Leaf	2016/6/28
HN8-1	C. siamense	Nanxingnongchang, Yalongwan, Sanya, Hainan	Jinhuang	Leaf	2017/4/17
HN10	C. siamense	Nanxingnongchang, Yalongwan, Sanya, Hainan	wild-unkown	Leaf	2017/4/17
HN18-1	C. siamense	Nanbinnongchang, Yacheng, Sanya, Hainan	Tainong	Leaf	2017/4/17
HN22-3	C. siamense	Ducun,Yacheng, Sanya, Hainan	Jinhuang	Leaf	2017/4/17
HN29-2	C. siamense	Haipo, Jiusuo, Ledong, Hainan	Tainong	Leaf	2017/4/17
HN39-2	C. siamense	No. 3 Century old Mango tree, Chahe, Changjiang, Hainan	wild-unkown	Leaf	2017/4/17
HN39-3	C. siamense	No. 3 Century old Mango tree, Chahe, Changjiang, Hainan	wild-unkown	Leaf	2017/4/17

HN50-2	C. siamense	Century old Mango tree, Gancheng, Dongfang, Hainan	wild-unkown	Leaf	2017/4/17
HN58-1	C. siamense	Hongxie, Yingchuan, Lingshui, Sanya, Hainan	unkown	Leaf	2017/4/17
SC2-1	C. siamense	Yakou, Yakou, Miyi, Sichuan	unknown	Leaf	2016/9/8
SC3-1	C. siamense	Shitou, Yakou, Miyi, Sichuan	unknown	Leaf	2016/9/8
SC3-2	C. siamense	Shitou,Yakou,Miyi, Sichuan	unknown	Leaf	2016/9/8
SC4-3	C. siamense	Shitou,Yakou,Miyi, Sichuan	unknown	Leaf	2016/9/8
SC5	C. siamense	Zaozilin,Yakou,Miyi, Sichuan	unknown	Leaf	2016/9/8
SC16	C. siamense	Ashuda, Panzhihua, Sichuan	Jilu	Leaf	2016/9/8
SC37-2	C. siamense	Zongfa, Renhequ, Panzhihua, Sichuan	Hongxiangya	Leaf	2016/9/8
SC38-1-1	C. siamense	Zongfa, Renhequ, Panzhihua, Sichuan	Hongxiangya	Leaf	2016/9/8
SC38-1-2	C. siamense	Zongfa, Renhequ, Panzhihua, Sichuan	Hongxiangya	Leaf	2016/9/8
SC38-1-3	C. siamense	Zongfa, Renhequ, Panzhihua, Sichuan	Hongxiangya	Leaf	2016/9/8
YN28-1	C. siamense	Hongxinfenchang, Namaluosizhai, Ganzhuang, Yuanjiang	Tainong	Leaf	2016/9/6
YN42-2-1	C. siamense	Minzufengqingyuan, Chengdong, Xishuangbanna, Yunnan	Yaomang	Leaf	2016/9/6
YN45-2-1	C. siamense	Chengdongdongwuyuan, Xishuangbana, Yunnan	unknown	Leaf	2016/9/6
YN47-2-1	C. siamense	Mangguosenlingongyuan, Yuanjiang, Yunnan	Tainong	Leaf	2016/9/6
YN56-1-1	C. siamense	Minzufengqingyuan, Xishuangbanna, Yunnan	Xiangya	Leaf	2016/9/6
