# Supplementary material for: Mating and Pathogenicity of the Dominant Colletotrichum Species Associated with Anthracnose Disease of Mango
Source: J Fungi (Basel). 2025 Oct 23;11(11):762. doi: 10.3390/jof11110762 (PMC12653461; doi:10.3390/jof11110762)
Supplement: Supplementary file 1 [file jof-11-00762-s001.zip › Table S5.pdf]

Table S5 Sequence variation in the ITS and ApMat loci among *C fructicola* parental isolates and their offspring

Isolate/Group	Number of polymorphic sites (Polymorphic site variation positions)	Comparison of polymorphic sites		
		Polymorphic sites variation position 1	Polymorphic sites variation position 2	Polymorphic sites variation position 3
		**Group 1**		
MG3-1	1 (611)	A	C	/
MG3-2	2 (611, 741)	A	T	/
YN21-1-3	/	C	C	/
GZ16	/	C	C	/
		**Group 2**		
MG4-1	0	C	G	/
MG4-2	2 (611, 691)	A	C	/
YN21-1-3	/	C	G	/
GZ21-2	/	C	G	/
		**Group 3**		
MG6-1	1 (540)	C	C	/
MG6-2	2 (540, 611)	C	A	/
HN19-1	/	G	C	/
FJ34-5	/	G	C	/
		**Group 4**		
MG7-1	1 (827)	C	/	/
MG7-2	1 (827)	C	/	/
HN19-1	/	A	/	/
FJ25-1	/	A	/	/
		**Group 5**		
MG8-1	2 (540, 611)	C	A	/
MG8-2	2 (540, 611)	C	A	/
HN19-1	/	G	C	/
FJ27-2	/	G	C	/
		**Group 6**		
MG10-1	1 (611)	A	/	/
MG10-2	0	C	/	/
FJ27-2	/	C	/	/
YN21-1-3	/	C	/	/
		**Group 7**		
MG11-1	0	C	/	/
MG11-2	1 (611)	A	/	/
FJ25-1	/	C	/	/
YN43-1	/	C	/	/
		**Group 8**		
MG12-1	1 (611)	A	/	/
MG12-2	0	C	/	/

FJ32-6	/	C	/	/
GZ2-1	/	C	/	/
		**Group 9**		
MG13-1	2 (540, 611)	C	A	/
MG13-2	2 (540, 611)	C	A	/
FJ26-1	/	G	C	/
FJ13-3	/	G	C	/
		**Group 10**		
MG14-1	3 (545, 546, 547)	C	T	T
MG14-2	0	-	-	-
HN7	/	-	-	-
GZ14-1	/	-	-	-

/ represents no data.

- represents the absence of a corresponding nucleotide.
